# Predictors of Health-Related Quality of Life in Patients at Risk for Cardiovascular Disease in European Primary Care

**DOI:** 10.1371/journal.pone.0029334

**Published:** 2011-12-22

**Authors:** Sabine Ludt, Michel Wensing, Joachim Szecsenyi, Jan van Lieshout, Justine Rochon, Tobias Freund, Stephen M. Campbell, Dominik Ose

**Affiliations:** 1 Department of General Practice and Health Services Research, University Hospital of Heidelberg, Heidelberg, Germany; 2 Scientific Institute for Quality of Healthcare, Radboud University Nijmegen Medical Centre, Nijmegen, The Netherlands; 3 Institute of Medical Biometry and Informatics, University Hospital of Heidelberg, Heidelberg, Germany; 4 Health Sciences – Primary Care Group, University of Manchester, Manchester, United Kingdom; Marienhospital Herne - University of Bochum, Germany

## Abstract

**Background:**

Cardiovascular risk management plays an important role in primary care. In patients at high risk for cardiovascular diseases (CVD) lifestyle and, where appropriate, medical interventions are recommended in guidelines. Health-related quality of life (HRQoL) is an important outcome in clinical practice. This study aimed to assess the HRQoL of this patient group and to investigate the impact of both patients' characteristics and practice quality scores on their assessments of HRQoL.

**Methods and Findings:**

An observational study in 218 general practices from 8 European countries was conducted. 2142 patients at risk for CVD (33.5% female) with a mean age of 66.3 (SD 9.1) years completed a questionnaire including the EQ-5D instrument and provided data from medical record. Validated quality indicators of general practices were assessed using practice questionnaires and face-to-face interviews. A hierarchical multilevel analysis was performed to identify predictors of EQ-5D scores at patient and practice level. The mean EQ-5D score was 0.78 (SD 0.19). Female gender (*r = −0.03*, *p<0.0016*), age (*r = −0.01*, *p = 0.0387*) and lower educational level (*r = −0.03*, *p<0.0001*) were correlated negatively with EQ-5D scores. Clinically more important was the correlation of HRQoL with the frequency of practice contacts (*r = −0.12*, *p<0.0001*) and the number of uncontrolled risk factors (*r = −0.01*, *p<0.0039*). Medication adherence (*r = 0.032*, *p<0.0001*), and physical activity (*r = 0.02*, *p<0.0001*) were identified as positive predictors of HRQoL. The EUPROPEP-score category ‘organization’ (*r = 0.02*, *p<0.0001*) was positively related to EQ-5D scores, whereas other practice scores were not correlated to EQ-5D-scores.

**Conclusions:**

In patients at risk for CVD, good medication adherence, regular physical activity, controlling of biomedical risk factor levels and patient-centered practice organization have been shown to be positively correlated to HRQoL and should therefore be targeted in interventions not only to reduce morbidity but also to sustain or even to ameliorate HRQoL.

## Introduction

Cardiovascular disease (CVD) and coronary heart disease (CHD) are major causes of premature death in Europe and also important causes of morbidity, contributing substantially to escalating healthcare costs [1]. CVD is a main contributor to the almost threefold difference in mortality between adult men and women in Europe [2]. Cardiovascular risk management (CVRM), which is mostly provided in primary care, includes counseling on lifestyle, preventive medication, where appropriate, and continuous monitoring to control modifiable risk factors such as high blood pressure [3], [4]. It has been stated that health services are predominately oriented towards care rather than prevention and towards acute rather than chronic care [2].

Previous research has focused on chronic care improvement resulting in the development of the chronic care model (CCM) [5], [6] that lead to health policy interventions to enhance evidence based chronic care such as incentivising chronic illness care in the United Kingdom [7] or the nationwide implementation of disease management programs in Germany [8]. However, research and policy efforts to improve risk management of individuals at risk for chronic diseases were less intensive, although the preventive impact is more considerable in this group [9].

As CVD develops usually over many years, general practitioners and general practice teams are in a unique position to provide continuous advice, support and counseling to patients at risk to prevent manifestation of CVD in a community setting [10]. Although there is given the opportunity to prevent CVD, especially in strong primary health care systems, where the general practitioner is acting as a gatekeeper, it has been shown that strong primary health care systems are more likely to make efforts to improve disease management but not necessarily efforts to improve delivery of lifestyle interventions [11].

HRQoL has gained increased attention as an outcome measure of interventions and treatments in patients with established cardiovascular disease [12], [13]. For individuals at risk for developing CVD, HRQoL measurement has been considered particularly useful because of two major reasons: As these individuals may be asymptomatic or have only mild symptoms over a long period of time, morbidity or mortality alone are insensitive measures of the impact of therapy, whereas HRQoL outcomes can help select therapeutic options [14]. Secondly, it may be difficult for these individuals to consider an asymptomatic illness as serious and to be aware of the benefit of medical treatment, especially if side effects of drugs may impair their life satisfaction [15].

There are different instruments available to measure HRQoL such as the SF-36 questionnaire and the EQ-5D instrument that has already been widespread used in CVD studies [12]. The EQ-5D instrument is a validated generic measure of health-related quality of life that was developed by the EuroQol Group [16] and it is simply for patients to understand and to complete.

There is a lack of studies that examine HRQoL in individuals at risk for CVD and also of studies that address predictors of HRQoL in this group [17]. The knowledge of these predictors may be useful to tailor interventions to the needs of individuals at risk for CVD aiming to improve both care and HRQoL. The aim of our study was therefore to describe HRQoL of individuals at risk for CVD in European primary care settings using the EQ-5D instrument and to identify predictors that have an impact on EQ-5D scores at patient and practice level.

## Methods

**Figure 1:**
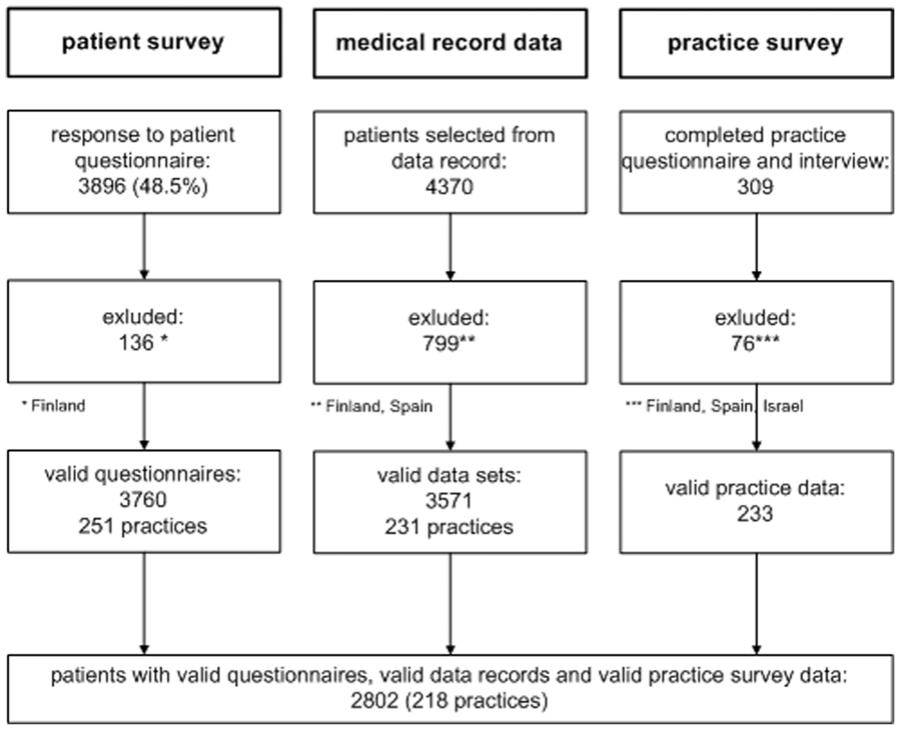
Data flowchart.

This study is part of the European Practice assessment (EPA) - Cardio project, focusing on the assessment of cardiovascular prevention and management in European primary care. In the first stage of the 4-year EPA-Cardio project (January 2006) we developed quality indicators to measure cardiovascular care [18] and identified additional instrument measures [19] including the EQ-5D for use in a subsequent observational study. The international cross-sectional observational study was conducted in 10 European countries between 2008 and 2009, i.e. Austria, Belgium, France, Finland, Germany, the Netherlands, Slovenia, Spain, Switzerland and the United Kingdom. In this part of the study Spain was excluded because only data from medical records were collected and Finland due to insufficient data quality. Israel was only involved in the practice survey (Figure 1). Ethics committees of all participating countries approved the study.

Full details of the study design and data collection methods have been published elsewhere [19], [20]. In summary, general practices were approached by the national research teams aiming to include a representative sample of 36 practices per country. Random samples of 30 patients at high risk for CVD per practice where identified from medical records according to the criteria listed in Table 1 and asked for participation, in order to receive informed written consent from at least 15 patients per practice (50% response).

**Table 1:** Inclusion and exclusion criteria.

Inclusion criteria	Exclusion criteria
1. High- risk patients defined by risk calculation with recommended tools according to national guidelines, e.g. 10% fatal CVD risk as calculated by the Dutch risk tables**or**2. Proxy measure: Patients with three out of the following four risk factors: hypertension, hyperlipidemia, smoking, men over 60 years	1. Patients with established CVD (including ischemic heart disease, myocardial infarction, angina pectoris, coronary surgery or revascularisation procedures, ischaemic stroke, transient ischemic attack, claudication or peripheral vascular disease)2. Patients with diabetes3. Terminal illness, cognitive disorders (e.g. dementia), psychiatric diseases (e.g. schizophrenia) and lack of language knowledge

### Measures

Patients were posted a questionnaire including demographic items (e.g. age, gender, education and marital status) and six validated survey instruments: These were 1) the ‘Rapid Assessment of Physical Activity (RAPA)’ [21], 2) the ‘Rapid Eating and Activity Assessment for Participants-Short Version (REAP-S) [22], 3) the ‘Mid-Sized model - baseline measurements for smoking [23], and 4) the 4-item Morisky-questionnaire to assess, where appropriate, medication adherence [24]. The questionnaire also included 5) the EUROPEP-instrument to evaluate general practice care [25], and 6) the EQ-5D instrument, that generates a single index score. It is based on a descriptive system that defines health in terms of the 5 dimensions ‘mobility’, ‘self-care’, ‘usual activities’, ‘pain/discomfort’ and ‘anxiety/depression’. The EQ-5D score has a range from 0 to 1(full health) and is calculated by applying scores from the EQ-5D preference weights elicited from the general population. For this study, the EQ-5D score was calculated using the value set for the European population [26], [27].

Additionally, patient data from medical records were collated using a paper based audit abstraction tool that included levels of blood pressure, cholesterol, body mass index (BMI) etc.

Researchers collated practice data by posting questionnaires and by face to face interviews with general practitioners using standardized interview guides. These instruments contained questions to characterize the practice according to size, location or number and function of practice staff. Quality indicators (QI) that were developed during the EPA-Cardio project [18] and derived from the EPA practice-management instrument [28] were converted into questions for the practice team. The quality indicators represented CVD care aspects (33 QI) [18] and organizational aspects of the practice management in the 3 dimensions ‘information process and technology’(11 QI), ‘organization of chronic care and prevention’ (19 QI) and ‘quality improvement’ (13 QI) [28]. All measures were piloted before being used in the study [19].

### Analyses

The main outcome measure was the EQ-5D score. To score practice quality indicators we aggregated the items of the practice questionnaires using the homogeneity analysis by alternating least squares (HOMALS). With this analysis, we identified 32 binary items with discrimination measures over 0.4 in two dimensions “practice quality management” (15 items) and “practice CVD care” (17 items) (Appendix S1). Scores were calculated by summing up the number of ‘yes’- answers resulting in a range from 0 to 15 for the quality-management score and from 0 to 17 for the CVD-care score.

Because of the hierarchical structure of the data, multilevel analysis was applied, which takes into account the dependence between patient outcomes (level 1) within primary care practices (level 2) nested within countries (level 3). Several models were evaluated treating practice and country levels as random effects and allowing explanatory variables at different levels (for details, see Table 2). The multilevel analysis started with an intercept-only (null) model for the three-level data without any predictor variables. Variance partition coefficients in each level were calculated using the restricted maximum likelihood (REML) method. The corresponding intra-class correlations (ICC) [29] at the practice and country level were provided. The next model included only the patient-level predictors as fixed effects. Finally, predictor variables on both patient and practice level were added as fixed effects. Explanatory variables on country level were not examined. In the final model adjusted for all variables, we included a total set of 13 potential explanatory variables, 11 on patient level and 2 on practice level (Table 2). The coefficients of the final model indicate the relation between the EQ-5D score and each explanatory variable. The differences between the “null”-model and the final adjusted model show to which extend the explanatory variables explain the variation in the outcome. Only patients for whose data on all explanatory variables on the different levels were available could be included in the final model. A non-responder analysis was performed between those patients included in the final dataset and those not included because of non-responding the EQ-5D items or other missing data. The significance level was set to 5% (two-sided). Regression coefficients and corresponding two-sided 95% confidence intervals (CI) were calculated and considered statistically significant if the CI excluded zero. All statistical analyses were carried out by using SPSS version 18.0 (SPSS Inc., Chicago, IL, USA). The multilevel analyses were conducted by using the procedure PROC MIXED in SAS version 9.2 (SAS Institute, Cary, NC).

**Table 2:** Explanatory variables included in the multilevel analysis.

Variables	Categories/Scoring
**Level 1: patient**	
Gender	2 categories: female; male
Age	Continuous: age divided by 5
Education	Years in school; 2 categories : ≤9 years; >9 years
Marital status	2 categories: married/cohabitating; single/separated/divorced/widowed
Frequency of practice attendance	Practice attendance within 12 months; 3 categories: up to 3 times/year; 4–7 times/year; more than 7 times/year
Medication adherence	Continuous sum score (Morisky- 4 items): 0–4 (best)
Physical activity	Continuous sum score (RAPA - 9 items): 1 (sedentary) -5 (regularly active)
Healthy diet	Continuous (REAP-S - 10 items:): mean: 1–3 (best)
Number of uncontrolled risk factors	Continuous sum score: 0–5: 1. mean RR >140/90 mmHg; 2. total cholesterol >5 mmol/l; 3. blood glucose (fasting>6.1 mmol/l or random >10 mmol/l); 4. BMI>30; 5. smoking
Patients' evaluation of practice care “clinical”	Continuous: (EUROPEP dimension ‘clinical behavior’ 16 items) mean: 1–5 (best)
Patients' evaluation of practice care “organizational”	Continuous: (EUROPEP dimension ‘organization of care ‘7 items) mean: 1–5 (best):
**Level 2: practice**	
Quality-management score	Continuous: sum score of ‘yes-answers’; range:0–15
CVD-care score	Continuous: sum score of ‘yes-answers’; range:0–17

## Results

### Demographics and practice characteristics

For 2802 individuals at high risk for CVD we were able to match data from medical records and survey instruments from 218 primary care practices in 8 European countries: Austria, Belgium, France, Germany, the Netherlands, Slovenia, Switzerland and the UK (Figure 1).

For 2554 individuals (91.1%) EQ-5D scores could be calculated. As we intended to examine the influence on medication adherence on EQ-5D scores we included only those patients who reported to take medication regularly reducing the number of included patients to 2318 (90.8% of responders). The sample size was reduced again due to missing data in the final multilevel hierarchical regression model with 13 potential explanatory variables to 2142. A non-responder analysis showed that excluded patients were similar to those included in most characteristics with exception of the following: They had less frequent practice contacts, were to a greater proportion smoker, and to a smaller proportion obese (BMI≥30) and had a slightly lower “healthy-diet” score. Furthermore, excluded individuals had higher EUROPEP scores. On average, 33.5% of the finally included patients were female and the mean age was 66.3 years. (SD 9.1). Most patients (79.0%) were married or cohabiting and had been in school for more than 9 years (68.3%). (Table 3)

**Table 3:** Patient characteristics (n = 2802).

	included (n = 2142)		not included (n = 660)		*P* *
**Age (years)**; mean (SD)	66.27	(9.07)	66.13	(9,82)	*0,739*
**Gender**					*0.925*
Female % (n)	33.5	717	33.2	219	
Male % (n)	66.5	1425	66.8	441	
**Marital status**					*0.372*
Single, separated, divorced, widowed % (n)	21.0	(450)	22.7	(139)	
Married, cohabited % (n)	79.0	(1692)	77.3	(474)	
**Education**					*0.099*
< = 9 years % (n)	31.7	(680)	35.5	(205)	
>9 years % (n)	68.3	(1462)	64.5	(373)	
**Frequency of practice attendance**					*0.000*
< = 3 times/year % (n)	34.1	(731)	48.0	(294)	
4–7 times/year % (n)	49.2	(1053)	36.7	(222)	
>7 times/year % (n)	16.7	(358)	15.7	(96)	
**CVD risk factors**					
RR >140/90 mmHg % (n)	50.9	(1036)	51.0	(346)	*1.000*
Cholesterol total > = 5 mmol/l % (n)	64.8	(1186)	69.2	(368)	*0.062*
Blood glucose fasting >6.1 or random >10.0 mmo/l % (n)****	14.9	249)	16.5	(84)	*0.399*
Smoker % (n)	23,0	(387)	33.1	(171)	*0.000*
BMI > = 30% (n)	31.0	(657)	28.2	(181)	*0.020*
**Number of uncontrolled risk factors** mean (SD)	1.64	(1.02)	1.68	(0.99)	*0.367*
**Lifestyle**					
Healthy diet** mean (SD)	2.25	(0.36)	2.18	(0.39)	*0.000*
Regular moderate physical activity % (n)	48.7	(1043)	53.0	(277)	*0.058*
**Medication adherence** (Morisky score = 4) % (n)	60.0	(1285)	54.8	(205)	*0.174*
**EUROPEP** ***					
Clinical behaviour; mean (SD)	4.50	(0.60)	4.59	(0.63)	*0.003*
Organisation of care; mean (SD)	4.46	(0.64)	4.52	(0.66)	*0.030*
**EQ-5D** mean (SD)	0.78	(0.19)	0.78	(0.20)	*0.688*

*p values are based on χ^2^ tests for categorical variables and on t tests for continuous variables.

**Maximum (best) = 3.

***Maximum (best) = 5.

****Although patients who were recorded as having diabetes were excluded, we asked to record blood glucose levels, because blood glucose measurement is recommend in guidelines for this patient group [3], [4].

In terms of risk factors, the majority of patients had increased levels for blood pressure, total cholesterol and blood glucose. Furthermore 23.0% were smokers and a third had a BMI of 30 or above. Half of the patients were underactive, defined as regular moderate (vigorous) physical activity less than 150 (60) min/week. The ‘healthy-diet’-score (maximum = 3 on a 3-point likert scale) was 2.2 (SD 0.36). EUROPEP scores reflecting patients’ evaluation of general practice care were over 4 on a 5-point likert scale ranging from 1(poor) to 5 (excellent), the subscale score ‘clinical behavior’ (4.50) was slightly higher than the subscale score ‘organization of care’ (4.46).

The included practices were mainly located in towns with less than 100,000 inhabitants with 2 full time equivalents (FTE) GPs and 1.5 FTE nurses in average. The 3 excluded practices showed no differences in relation to these characteristics. Means of practice quality-scores were 8.3 (SD 3.62) for quality management (range 0–15) and 8.19 (SD 4.41) for CVD care (range 0–17).

### EQ-5D scores and predictors of EQ-5D levels

The overall mean EQ-5D score was 0.78 (SD 0.19). In the multilevel analyses the intercept-only model showed that the greatest proportion of variance in EQ-5D scores occurred at the patient level (92.3%). The proportion of variance (intra-class coefficients -ICC) at practice level was estimated to 2.7%, .the proportion of variance at practice level was estimated to 5.0%.

Including explanatory patient variables into the model resulted in smaller variance proportions, meaning that these variables explained the variance. Additional including of explanatory practice variables resulted in the final adjusted model that explained the variance at the country level to 23%, at the practice level to 50% and the variance at the patient level plus random to 14%. Table 4 provides details of the relationship between the explanatory variables on patient and practice levels respectively and the EQ-5D scores. Adjusted for all other variables, regression coefficients indicate the changes of the EQ-5D score in comparison to a baseline category for categorical variables or with one unit increase of a continuous variable. At patient level, EQ-5D index scores of female patients were lower compared to male patients (*r = −0.03*; *p = 0.0016*). Each 5-year increase in age was associated with a 0.01 decrease in EQ-5D scores (*r = −0.01*; *p = 0.0387*). Patients with a lower educational level scored HRQoL lower than higher educated individuals (*r = −0.03*; *p<0.0001*). Each uncontrolled risk factor (e.g. mean blood pressure level over 140/90) was associated with a 0.01 decrease in EQ-5D scores (*r = −0.01*; *p = 0.0039*) resulting in a maximum difference of 0.05 between patients with zero and five uncontrolled risk factors. Each increase of one point of the “Morisky score” (0–4) indicating medication adherence was linked to a 0.02 increase of EQ-5D scores (*r = 0.02*; *p<0.0001*) resulting in a maximum increase of 0.08 (4×0.02) for the highest score (4) compared with the lowest one (0). Increasing physical activity levels were related to higher ratings of HRQoL (*r = 0.02*; *p<0.0001*) with a 0.02 increase of EQ-5D scores per unit.

**Table 4:** Parameter estimates of the final multilevel model with overall EQ-5D score as dependent variable (N = 2142 patients. 215 practices. 8 countries).

	Regression coefficient	Standard error	95% confidence intervals	*P value*
**PATIENT CHARACTERISTICS**				
Age				
Continuous (5-year units)	−0.01	0.00	[−0.02; −0.00]	*0.0387*
Gender				
Female	−0.03	0.01	[−0.05; −0.01]	*0.0016*
Male	Reference			
Education. Years in school				
<9 years	−0.03	0.01	[−0.04; −0.02]	*<0.001*
>9 years	Reference			
Marital status				
Married, cohabitating	−0.01	0.02	[−0.04; 0.02]	*0.5741*
Single, separated, widowed	Reference			
Frequency of practice attendance				
Up to 3 times per year	0.12	0.02	[0.08; 0.15]	*<0.001*
4–7 times per year	0.06	0.01	[0.04; 0.09]	*<0.001*
more than 7 times per year s	Reference			
Medication adherence (Morisky)				
Continuous	0.02	0.00	[0.01; 0.03]	*<0.001*
Physical activity status				
Continuous	0.02	0.00	[0.01; 0.03]	*<0.001*
Healthy diet (score)				
Continuous	0.02	0.01	[−0.00; 0.03]	*0.0960*
Number of uncontrolled risk factors				
Continuous	−0.01	0.00	(−0.01; −0.00]	*0.0039*
EUROPEP score ‘clinical behavior’				
Continuous	0.01	0.01	[−0.01; 0.02)	*0. 2199*
EUROPEP score ‘organization of care’				
Continuous	0.02	0.00	[0.02; 0.03]	*<0.001*
**PRACTICE CHARACTERISTICS**				
Practice quality management				
Continuous	0.00	0.00	[−0.00; 0.00]	*0.0904*
Practice CVD care				
Continuous	0.00	0.00	[−0.00; 0.00]	*0.6529*

Married or cohabiting individuals had higher EQ-5D scores than singles but this relationship was not significant (*r = −0.03*; *p = 0.5741*). Although patients' evaluation of organizational aspects of practice care (EUROPEP score ‘organization of care’) was linked markedly and significantly (*r = 0.02*; *p<0.0001*) to HRQoL, namely 0.02 increase of EQ-5D scores with one increasing unit of the EUROPEP score (0–7), quality scores of general practice performance were not significantly associated. (Table 4).

## Discussion

Our study has 3 main findings: Firstly, health-related quality of life (HRQoL) is impaired in patients at risk for cardiovascular diseases. Secondly, HRQoL is correlated to patient characteristics with limited practical relevance. The third and most clinically important finding is that we identified positive predictors of HRQoL; namely good medication adherence, regular physical activity, control of modifiable risk factor levels and providing patient-centered organizational practice support. These predictors have also substantial scope to reduce morbidity and mortality and should therefore be focused in efforts to improve prevention of CVD.

Relating to our first result, the mean EQ-5D score was 0.78 for individuals at risk for CVD. It has been shown that this level is in line with findings from other CVD-studies describing similar scores for Non-CHD individuals over 65 years [17] and equal scores for CHD-patients in mild disease stages in comparison to lower scores (0.51) for CHD-patients in severe disease states [12].

Regarding socio-demographic predictors of the health-related quality of life, we found a negative relationship between patient characteristics, such as female gender, increasing age and lower educational level, and HRQoL. These findings were also reported in patients with established CVD in previous research [17], [30]–[34]. In terms of the practical impact of our study results, most variables at the patient level reached statistical significance in the final 3-level model, but because of the large sample size in our study, we should also consider what is clinically relevant. Earlier studies on clinically relevant differences using EQ-5D index score defined a mean minimally important difference for the EQ-5D of 0.074 (range: 0.011–0.140) [35]. This means that the two categorical variables gender and educational level with regression coefficients (*r*) of 0.03 might be less clinically important. The relationship between the continuous variable “age” has to be interpreted as changes in the EQ-5D index scores of −0.01 per unit (5 years), meaning that only a difference of 35 years in age (7×5) is related to a clinically important difference of HRQoL, as measured by a difference in EQ-5D index scores of 0.07. These findings indicate that only large differences in age are negatively correlated with HRQoL and that smaller differences in age (e.g. between 40–50 years) are not clinically important correlated with impaired HRQoL.

A more clinically important positive predictor of HRQoL was physical activity. In our study, increasing physical activity ranging from sedentary (0) up to a regular moderate (vigorous) physical activity of at least 150 (60) min per week (5) was significantly associated with increased EQ-5D levels in individuals at risk for CVD. As the RAPA instrument defines 5 degrees of physical activity, the *r* of 0.02 indicates that there is a maximum increase of 0.1 (5×0.02) comparing EQ-5D index levels of sedentary individuals to regular active ones (Table 4). This can be regarded as a clinically important amelioration of HRQoL. A positive relationship between physical activity and HRQoL was also found in previous research for both populations with established diseases [36] and general adults [37]. Sedentary lifestyle has been reported as to be the most prevalent risk factor [38], [39]. Persons older than 60 years appear to benefit from exercise training at least as much as younger adults, and regular physical activity can reduce the risk of CHD and extend the active lifespan [40]. Additionally it is reported that lifestyle interventions especially on physical activity ameliorate HRQoL [31].

Therapeutic encouragement of regular physical activity should therefore be a major aim in the prevention of CVD, especially in groups of high risk patients.

The number of uncontrolled (not achieving treatment goals) risk factors (RR mean >140/90, cholesterol total >5 mmol/l, blood glucose >6.1 mmol - fasting or >10 mmol-random, BMI>30 and smoking) were associated with impaired HRQoL in our study. The *r* of −0.01 indicates, however, that this relationship may be weaker than the relationship between physical activity and HRQoL: In comparison, HRQoL of individuals with uncontrolled levels of risk factors (RR, cholesterol, blood glucose) who were obese and smokers, was only impaired minimally (decrease of 0.05 in EQ-5D levels) compared with individuals without any of these risk factors number. From the view of GPs, it is desirable that better risk factor control is associated with improved HRQoL. From the patient perspective, however, these risk factors are normally asymptomatic or they feel even worse under treatment [14]. Medical treatment can be accompanied by side-effects of drugs that may impair life satisfaction [15].

Previous studies have shown that risk factors among individuals at risk for CVD are poorly controlled [41]. For practical reasons, it is therefore important to focus on patients' handling of medical treatment to prevent poor medication adherence due to side effects and impaired HRQoL. The positive relationship between risk factor control and HRQoL that was found in our study shows that it is possible to control risk factor levels without impairing HRQoL. Our findings rather suggest that it is possible not only to reduce morbidity but also to improve HRQoL slightly by controlling risk factors.

This result resonates with an additional result of our study, namely that increasing medication adherence (Morisky score) was associated with higher EQ-5D scores. This result can also be regarded not only as statistically but also as practically significant, as there is a 0.08 difference in EQ-5D index scores between the lowest degree of the Morisky score indicating poor medication adherence, and the highest score indicating best medication adherence. A recently published study reported a positive impact of HRQoL on medication adherence in hypertensive adults over 65years [42]. The direction of this relationship remains unclear und might be investigated in further research, but it seems that HRQoL and medication adherence are correlated positively in both directions.

It has been stated to consider medication nonadherence as an unrecognized cardiovascular risk factor [43] and it has been reported that nonadherence is associated with increased risk for all cause mortality, cardiovascular mortality and also with cardiac-specific outcomes, such as hospitalization, heart failure and coronary revascularization procedures, in patients with CHD [44]. This may channel the focus on strategies to promote medication adherence, such as continuous monitoring, decreasing dose frequency, motivational approaches or combined strategies [45], [46] especially in patients at high risk for CVD who normally require lifelong treatment and support [47].

The frequency of practice contacts per year was also related to lower EQ-5D scores in our study. There is a clinically important relationship between the frequency of practice consultations and HRQoL. The HRQoL of patients attending the practice (GP) only up to 3 times a year (EQ-5D index score 0.12 greater), or more frequently up to 7 times a year (EQ-5D index score 0.06 greater) is improved compared to patients visiting the practice more than 7 times a year. The frequency of practice contacts per year may also reflect the severity of disease and be therefore associated with lower EQ-5D scores [12].

Practice quality indicators u in our study did not show any statistically or clinically significant associations with patients' HRQoL in patients at risk for CVD. Maybe, other indicators relating to the personal interaction between GP and patient or service aspects would have been more suitable to reflect preventive care in this patient group.

However, it was possible in our study to show the impact of practices' organizational management on patients' HRQoL regarding predictors that were assessed at the patient level to assess satisfaction with care, as the variables included at this level explained 48.1% of the variance at the practice level: One part of the EUROPEP instrument (7 items) evaluates patients' satisfaction with organizational aspects of care, such as waiting times, helpfulness of the practice staff, ability to speak the GP on the phone etc. The regression coefficient, calculated in the multilevel analysis, indicates a 0.02 increase of EQ-5D scores with each unit increase of the organizational score of the EUROPEP instrument. This means a possible maximum increase of EQ-5D scores by 0.14 (0.02×7). This result identifies patient-centered organizational aspects of practice management as clinically significant positive predictors of HRQoL.

### Strengths and limitations

The EPA cardio study is one of the largest international studies on the management of cardiovascular prevention in European primary care [20]. We used multilevel modeling to identify predictors of health-related quality of life in one model adjusting for all other variables. Hierarchical models combine information across units to produce accurate and well calibrated prediction of outcomes [48]. This analytic approach has been seen to be very relevant in health services research as patients' data were similarly clustered at more than one level [49]. We used validated measures and collected morbidity data from medical record in contrast to self reported morbidity indicators that could lead to misclassifications.

Nevertheless, in some countries it was difficult to enroll 36 practices as intended and different sampling methods were used to identify individuals at high risk for CVD, i.e. by risk calculation with recommended instruments or by identifying the presence of risk factors. In the multilevel analyses, the total number of cases decreased due to missing data, as we conducted a complete cases analysis. The EQ-5D instrument showed a ceiling effect with 30% of people scoring the highest value. As also reported in other studies, EQ-5D may be less sensitive to describe mild-severity health levels. However, the EQ-5D instrument is reported to have a better discrimination capacity for socio-demographic and morbidity indicators that were focused in our study [50]. Because of the observational design of our study, the correlations found cannot be used to attest causal associations.

### Conclusions

Our study results suggest that HRQoL, as an important patient related outcome in patients at risk for CVD is correlated statistically but less clinically significant with socio-demographic factors such as age, gender or educational level. Additionally, healthy behavior, such as regular physical activity and a good medication adherence, and also organizational aspects of practice management were identified as clinically important predictors of improved HRQoL. Controlling of risk factors in asymptomatic individuals is possible without impairing HRQoL. It seems to be possible to improve both reducing morbidity and ameliorating HRQoL by interventions that focus on medication adherence, treatment of modifiable risk factors and lifestyle counseling, especially to increase physical activity. Patient-centered organization of practice management may also play an important role for ameliorating HRQoL of patients at risk for CVD. On the other hand, it seems to be important that medical or behavioral treatment to control risk factors in asymptomatic individuals, which require lifelong treatment and counseling, should be careful to address patients' HRQoL to prevent poor treatment adherence due to impaired HRQoL. Research and policy might focus on the development and implementation of “risk management programs” including these key elements to prevent CVD more effectively.

## Supporting Information

Appendix S1 **Practice quality indicators.**
(DOC)
