# Supplementary material for: Predictors of Health-Related Quality of Life in Patients at Risk for Cardiovascular Disease in European Primary Care
Source: PLoS One. 2011 Dec 22;6(12):e29334. doi: 10.1371/journal.pone.0029334 (PMC3245263; doi:10.1371/journal.pone.0029334)
Supplement: Appendix S1 — Practice quality indicators. (DOC) [file pone.0029334.s001.doc]

**Appendix S1: Practice quality indicators**

- Quality management (15 items)
- Does the practice use a computer-supported patient file system?
- Is the computer used for creating medication prescriptions?
- Does the practice have a procedure for the management of patient information in relation to detailed examination results and the documentation of measures that were taken (e.g., blood examinations)?
- Does the practice have a procedure for the management of patient information in relation to the review of detailed examination results by the doctor (in terms of outgoing needs)?
- Do the practice doctors have direct access to medical guidelines (either on paper or electronic) in their treatment rooms?
- In general: Is practice staff allowed to contact or recall patients?
- Does the practice produce a quality report?
- Has the practice undertaken at least one clinical audit in the last 12 months?
- Did you set standards regarding this clinical audit (defined the target)?
- Did you collect data regarding this clinical audit?
- Did you evaluate the result?
- Were you able to improve the quality regarding this clinical audit topic?
- Does the practice have a critical incident register?
- Did the practice have a team meeting about quality improvement relating to CVD at least once in the last 15 months?
- Did the practice participate in cardiovascular quality improvement projects?
- CVD care (17 items)
- Does the practice use case finding methods to detect patients with cardiovascular risk factors?
- Does the practice use a system for recalling patients with cardio vascular diseases?
- Does the practice use a system for recalling patients with diabetes?
- Does the practice use a system for recalling patients with hypertension?
- Does the practice use a system for recalling populations at risk for preventive care regarding cardio vascular diseases?
- Does the practice use a system for recalling populations at risk for preventive care regarding influenza?
- Does the practice have a procedure for smoking cessation (e.g. with the Minimal Intervention Strategy)
- Does the practice participate in public health care programmes on life style (physical exercise, stop smoking)?
- Did all nurses attend ≥ one training/continuing medical education event on CVD within the last 5 years?
- Did nurses take part in local/community campaigns or actions on CVD risk prevention (e.g. stop smoking campaigns, fun-runs etc)?
- Does the practice use a CVD standardized risk assessment tool?
- Is the CVD risk assessment tool integrated with the patient medical record system (e.g. so that the CVD event risk score is entered directly in to the patient's medical record)
- Is there in general a record in the electronic or paper based patient record that the CVD standardized risk assessment tool has been offered?
- Is CVD risk advice (e.g. about modifiable risk factors such as diet and exercise) integrated with the patient medical record system?
- Do you offer regularly two or many consultations to provide advice on patient’s life style?
- Does the practice have an up-to-date directory of prevention activities/organizations available locally (e.g. gyms, walking group, weight-watchers etc)?
- Did your practice participate in a project concerning cardiovascular risk management the last 2 years (apart from those mentioned above)
